# Dietary Lipid Profile in Spanish Children with Overweight or Obesity: A Longitudinal Study on the Impact of Children’s Eating Behavior and Sedentary Habits

**DOI:** 10.3390/nu17030494

**Published:** 2025-01-29

**Authors:** Silvia García, Marina Ródenas-Munar, Emma Argelich, David Mateos, Lucía Ugarriza, Josep A. Tur, Cristina Bouzas

**Affiliations:** 1 Research Group on Community Nutrition & Oxidative Stress, University of the Balearic Islands-IUNICS, 07122 Palma de Mallorca, Spain; silvia.garcia@uib.es (S.G.); davidfrom13@gmail.com (D.M.); luciaugarriza@gmail.com (L.U.); cristina.bouzas@uib.es (C.B.); 2 CIBEROBN (Physiopathology of Obesity and Nutrition), Instituto de Salud Carlos III, 28029 Madrid, Spain; 3 Health Research Institute of Balearic Islands (IdISBa), 07120 Palma de Mallorca, Spain; 4 Camp Redó Primary Health Care Center, 07010 Palma de Mallorca, Spain

**Keywords:** childhood obesity, screen time, eating behavior, dietary lipids, saturated fat, nutrition education

## Abstract

Background: Dietary lipids, sedentary habits, and eating behaviors influence childhood obesity, but their interrelations remain unclear. Aim: To assess the relationships between young children’s dietary lipid profile and children’s eating behavior, as well as their sedentary habits, providing evidence-based insights that can help mitigate obesity risk in this vulnerable population. Design: A longitudinal randomized controlled trial conducted over 9 months, involving 90 children aged 2–6 years with overweight or obesity who were followed under a program that promoted healthy lifestyle habits for all participants and regularly monitored their parameters. Methods: The dietary lipid profile, eating behavior, and sedentary habits were assessed at baseline and after 9 months using validated tools, including 24 h recalls, the child eating behavior questionnaire (CEBQ), and screen time questionnaires. Results: Reductions in screen/sedentary time were significantly associated with decreased total fat (−11.1 g/day) and saturated fat (−4.3 g/day) intake, compared to smaller reductions in unchanged screen hours and increases with prolonged screen use. A positive correlation was observed between changes in dietary fat and the CEBQ domain “Desire to drink” (r = 0.528, *p* < 0.001), with regression analysis confirming a direct relationship. Conclusions: Managing screen time and eating behavior is crucial for improving dietary lipid profile and reducing childhood obesity risk. Strategies should include reducing sedentary behaviors, limiting sugary drinks, and promoting water as the main beverage, alongside effective nutrition education for families.

## 1. Introduction

Childhood obesity has reached alarming levels globally; it is estimated that over 390 million children and adolescents aged 5–19 years old were overweight or obese in 2022, and that 37 million children under the age of 5 years old are overweight [1]. Projections indicate that the prevalence of childhood obesity is expected to continue to rise, with estimates suggesting that, if current trends persist, most (57.3%) of today’s children will be obese by adulthood. Additionally, forecasts predict that by 2050, approximately one-third of adolescents will have obesity [2,3]. This rising trend presents not only immediate health risks but also long-term consequences including an increased likelihood of developing non-communicable diseases such as type 2 diabetes, cardiovascular disease, and certain cancers [1]. Addressing this public health crisis demands a comprehensive understanding of factors contributing to obesity and related metabolic disorders.

Among the determinants of childhood obesity, dietary habits play a critical role [4,5]. In particular, dietary lipid profile, which encompasses the range of fats consumed, including saturated fats (SFAs), monounsaturated fats (MUFAs), and polyunsaturated fats (PUFAs), are essential indicators of metabolic health [6]. Research has reported that an imbalance in lipid intake, characterized by excessive consumption of SFAs and trans fats (TFAs), and an insufficient intake of healthy fats such as MUFAs and PUFAs, including omega-3 fatty acids, can lead to adverse health outcomes, influencing not only weight gain but also lipid metabolism and overall cardiometabolic health [7]. The World Health Organization (WHO) recommends, for both adults and children, to reduce SFA intake to 10% or less of the total energy intake, and to reduce TFA intake to 1% or less of the total intake, while encouraging the consumption of MUFAs and PUFAs [7,8,9]. The factors that shape lipid profile in young children are complex and multifaceted, demanding a nuanced examination of several lifestyle and behavioral components [5].

Children’s eating behaviors are shaped by a variety of intrinsic and extrinsic factors, including their preferences, appetite regulation, and responses to environmental cues [10]. These behaviors influence food choices, portion sizes, and overall dietary patterns, which are critical in shaping their nutritional status and health outcomes [11]. Research highlights the importance of understanding how specific eating behaviors, such as food neophobia, emotional eating, and satiety, can affect dietary quality, energy balance, and nutrient intake [10,11,12,13,14]. However, the relationship between eating behaviors and dietary lipid profiles remains underexplored.

In addition to eating behavior, sedentary behavior, particularly associated with screen time and a lack of physical activity, are increasingly recognized as significant risk factors for obesity and poor metabolic health in children [15]. Screen time has become a dominant component of sedentary behavior in childhood currently, contributing to reduced physical activity and prolonged periods of inactivity [16]. Excessive screen exposure has also been associated with disrupted sleep patterns and a higher likelihood of developing poor eating habits, such as snacking on energy-dense, nutrient-poor foods during screen use, influenced by factors such as exposure to unhealthy food advertisements, distraction during meals, or the deregulation of hunger and satiety cues [17,18,19]. As young children spend considerable time engaged in these sedentary activities [20], it is imperative to explore how these habits interact with eating behaviors and impact dietary lipid profile.

In recent years, research on childhood obesity has increasingly focused on the role of dietary habits, sedentary behaviors, and their combined influence on health outcomes. There is existing literature focusing on the relationship between sedentary behavior and physical activity with plasma lipid profiles but not diet lipid profiles [21,22]. Some studies have explored dietary quality in relation to these same parameters, showing that lower sedentary behavior and higher physical activity levels are associated with greater consumption of vegetables, fruits, and healthy fats [23,24]. However, research specifically addressing the impact of sedentary behavior and eating habits on dietary lipid profile remains limited. Therefore, this study aims to address this gap, providing novel insights into how these factors influence the lipid composition of children’s diets.

Moreover, the majority of studies examining the impact of these variables together have focused on older children or adolescents at school age [25,26,27]. While valuable, these studies do not fully capture the complexities and developmental nuances present in younger children, particularly those aged 2 to 6 years. This age group is crucial for understanding early-life factors that could potentially influence lifelong health trajectories, including the development of metabolic disorders such as obesity and dyslipidemia. Limited studies have begun to explore the link between sedentary behavior and dietary intake in preschool-age children, emphasizing the importance of early interventions. For example, it has been shown that prolonged screen time in young older children is associated with an increased intake of high-calorie, low-nutrient foods, which may contribute to poor dietary lipid profiles [28,29]. Research suggests that the combination of sedentary behaviors and unhealthy eating habits in early childhood may have a compounding effect on metabolic health, including the regulation of lipid metabolism and body composition [30]. However, few studies have directly linked changes in both screen time and children’s eating behavior to alterations in dietary lipid profiles in younger children.

This study aims to assess the relationships between young children’s dietary lipid profile and children’s eating behavior, as well as their sedentary habits. Understanding these independent relationships is crucial for developing evidence-based strategies to mitigate obesity risk in this vulnerable population [1]. By exploring these connections, this study seeks to provide valuable insights into the existing literature on childhood obesity prevention.

## 2. Methods

### 2.1. Design

This longitudinal study employed a two-arm parallel randomized controlled trial design to evaluate interventions for overweight and obesity in children. After initial baseline evaluations, participants were allocated to either the intervention or control group in a 1:1 ratio. Both groups received general healthy lifestyle recommendations; however, the intervention group participated in a 10-week parental support program, followed by a validated 6-month e-health initiative, while the control group received standard general health recommendations. Further details can be found in the study protocol [31]. In the present analysis, the sample was treated as a single cohort. This analysis was conducted within the framework of the main study but was treated as a cohort to align with the primary focus of evaluating longitudinal changes across the entire sample, rather than conducting group-specific comparisons.

### 2.2. Participants, Recruitment, and Ethics

A total of 90 families were included in the present study. To be included, children had to be between 2 and 6 years old and be overweight or obese according to the international obesity task force (IOTF) cut-offs. [32]. The exclusion criteria were children with any underlying medical conditions, those already receiving treatment for overweight or obesity, or whose parents were unable to communicate in the local language, and families without access to a smartphone. Assessments occurred at baseline and after 9 months. Recruitment followed a standardized process [33].

Families visiting pediatricians for routine height and weight checks at primary care centers or hospitals were invited to participate. If the parents expressed an interest, the pediatrician scheduled a follow-up meeting within 7 days to provide further study details and obtain informed consent. All families who agreed to participate in the study returned a signed consent form. A member of the research team co-signed the document, and a copy was provided to the family. Following this, an appointment for baseline assessments was scheduled. The study protocol adhered to the SPIRIT 2013 guidelines and to the principles of the Declaration of Helsinki. All procedures were approved by the Ethics Committee of the Balearic Islands (Palma de Mallorca, Spain, ref. IB 3814/18 PI; 13 February 2019).

### 2.3. Sociodemographic Characteristics and Anthropometric Measurements

The sociodemographic characteristics collected included the child’s age, sex, and the parental education level, as well as family income, employment status, and household composition. These data were gathered through structured questionnaires completed by the parents during baseline assessments.

The children’s weight and height were recorded to the nearest 0.1 kg and 0.1 cm, respectively. Height was measured using a fixed stadiometer, and weight was assessed with the children wearing only underwear, and the head on the Frankfurt’s plane. Body mass index (BMI) was calculated by dividing weight (in kilograms) by the square of height (in meters). Waist circumference was also measured; taken at the midpoint between the lowest rib and the iliac crest, using a non-elastic measuring tape to the nearest 0.1 cm. Body weight, height, and waist circumference, were taken three times, and the means of these measurements were used for analysis. All measurements were performed at baseline and after 9 months by trained healthcare professionals in a standardized manner, using calibrated equipment to ensure accuracy and consistency.

### 2.4. Children’s Eating Behavior

The children’s eating behavior was evaluated using the Child Eating Behavior Questionnaire (CEBQ) [34], a validated tool that assesses various eating styles through 35 items grouped into eight factors associated with obesity risk [34,35]. The specific eight factors are: food responsiveness, the enjoyment of food, emotional overeating, the desire to drink, satiety responsiveness, slowness in eating, emotional undereating, and fussiness. Parents rated each question on a five-point Likert scale, with responses ranging from “never” to “always” for the first 13 items, and from “disagree” to “agree” for the remaining items. Mean scores were calculated for each subscale to provide a comprehensive profile of the child’s eating habits, and differences in the results between baseline and 9 months were also calculated.

### 2.5. Screen Time—Sedentary Behavior

Sedentary behavior is defined as any waking behavior performed in a sitting, reclining, or lying posture, excluding sleep. It typically includes activities such as screen use, reading, or sitting during transportation, and has been associated with various adverse health outcomes, particularly when prolonged [36].

Sedentary behavior was assessed through parent-reported data on the children’s screen time and household screen device availability. Parents were asked to report the average daily time their child spent engaging in screen-based activities (e.g., watching TV, using tablets, smartphones, or computers) on both weekdays and weekends. Additionally, they provided information on the total number of screen devices available in the household, including televisions, computers, tablets, and smartphones. These data were collected to estimate the children’s overall screen exposure and to quantify sedentary behavior across different contexts within the home environment. Each child’s screen time per week was determined, and differences in screen time between baseline and 9 months were also calculated.

### 2.6. Dietary Lipid Profile: 24 h Recall

Participants were instructed on how to complete a three-day 24 h dietary recall (24 h) during an in-person interview. The 24 h is often used as a gold standard method in dietary assessment [37]. The recalls were completed on-site with guidance from a dietitian, who reviewed them immediately for accuracy and completeness. Two of the recalls focused on weekday consumption (e.g., Monday to Thursday), while one covered a weekend day (e.g., Friday, Saturday, or Sunday). Every 24 h, detailed information was collected on mealtimes, locations, the types of food consumed, and portion sizes, which participants could express using common household measurements (e.g., cups, teaspoons, tablespoons) or provide weights in grams (g) and volumes in milliliters (mL). Energy, macronutrient, and micronutrient intakes were calculated by multiplying the frequency of consumption by the nutrient content of the specified portion sizes for each food item. Daily intake values were derived by dividing these results by the seven days of a standard week.

The 24 h data were processed using EvalFINUT^®^ version 2.0 (FINUT, Granada, Spain), a program based on the Spanish Food Composition Database (BEDCA) and the USDA food database. This software converted food consumption data into dietary intake values for macronutrients and micronutrients. The mean values from the three 24 h periods were used as a reference for dietary intake assessments, with results presented as daily consumption in kilocalories (kcal) for energy and grams (g), milligrams (mg), or micrograms (µg) for nutrients, with special emphasis on analyzing the lipid profiles of the diet, as this was the primary objective of the study. Differences between lipid profiles at baseline and 9 months later were calculated.

### 2.7. Statistics

The analysis was conducted using the SPSS statistical software package version 27.0 (SPPS Inc., Chicago, IL, USA). Data are presented as mean and standard deviation (SD), except for prevalence data, which are expressed as sample size and percentage. Group differences were tested with the Chi-squared test for categorical variables, while one-way ANOVA was applied to continuous variables. Changes in weekly screen hours from baseline to 9 months were categorized into tertiles: Tertile 1 (T1) represented a reduction in screen/sedentary time (<1.5 h of weekly screen reduction), Tertile 2 (T2) represented a group without changes in screen/sedentary time (−1.4 through 0.7 weekly screen hours change), and Tertile 3 (T3) represented an increased screen/sedentary time (>0.8 h of weekly screen increase).

The General Linear Model (GLM) was used to assess the relationships between changes in screen/sedentary time and dietary fat parameters, such as total fat (g), SFA (g), MUFA (g), PUFA (g), cholesterol (mg), eicosapentanoic acid (g) or EPA, docosahexaenoic acid (g) or DHA, total energy intake (kcal), and fiber (g), over a 9-month period, with adjustments for the socioeconomic status of the family and the children’s BMI (kg/m^2^), weight (kg), and height (m). Bonferroni’s post hoc test was applied to identify statistically significant differences between groups (*p* < 0.05). Time-related changes within the group are represented with “a, b, c”, letters denoting group differences at each time point (ANOVA). Moreover, the letters “d, e, f” denote intergroup differences in time-related changes (post hoc analysis). Pearson correlations were calculated between changes in the children’s eating behavior (analyzed with the CEBQ) and changes in dietary fat within a 9-month period. A linear regression analysis was performed for the Pearson significant variables, examining the relationship between changes in the desire to drink and changes in dietary fat within a 9-month period, with dietary fat being the dependent variable.

## 3. Results

Table 1 shows the sociodemographic characteristics of the sampled children (n = 90). There were more girls (66.7%) than boys (33.3%), and the mean age and BMI were 5.3 years old and 23.6 kg/m^2^, respectively.

Table 2 shows differences in the dietary fat profile between baseline and 9 months related to changes in screen/sedentary time (measured by weekly screen hours, which includes week and weekend screen hours). The results show how a reduction in screen/sedentary time is significantly related to dietary total fat (or total fat consumed in a daily diet). Reductions of −11.1 g of total fat are seen in T1 (the group which has decreased its screen/sedentary time) compared to −6.9 g of total fat reduced in T2, and to an increase of 8.1 g of total fat in T3 (the group which has increased its screen/sedentary time). Similar results are shown in dietary SFA with higher reductions in SFA in T1 (−4.3 g) compared to T2 (−2.4 g) and compared to an increase in SFA in T3 (2.1 g). Other fat profile variables showed non-statistical significance.

**Figure 1:**
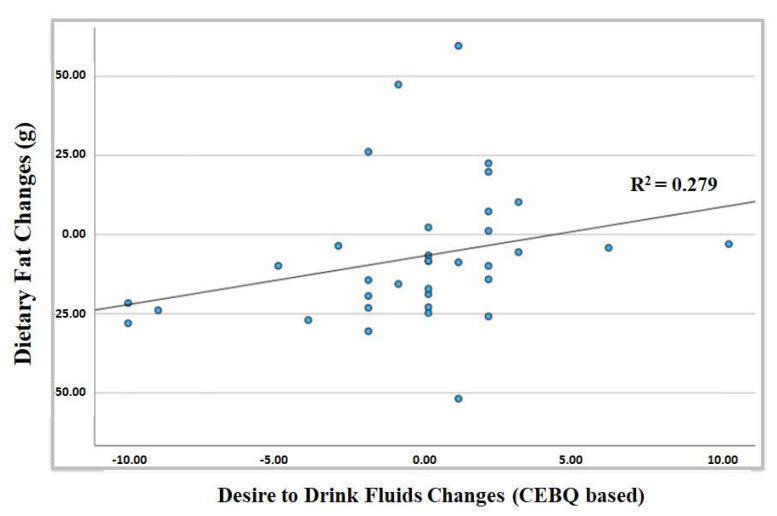
Scatter plot graphic and regression line between changes in the desire to drink fluids and changes in dietary fat within a 9-month period.

Pearson correlation analysis was performed between changes in dietary fat and changes in children’s eating behavior (evaluated by CEBQ), and it is shown in Table 3. The CEBQ is divided into 8 domains, as shown in Table 3, and the changes in each domain were evaluated individually to assess their relationship with changes in dietary fat within a 9-month period. The analysis showed how changes in dietary fat have been related with the “Desire to drink” domain with values of *r* = 0.528 and *p* = <0.001. Finally, a linear regression analysis was performed between the Pearson statistical significative variables, relating changes in dietary fat and changes in the children’s desire to drink fluids, between baseline and a 9-month period. This linear regression analysis can be seen in Figure 1, showing the children’s increased desire to drink fluids and their higher dietary fat, which shows a direct relationship between changes in the children’s desire to drink and changes in dietary fat or fat intake in a daily diet (*p* < 0.001).

## 4. Discussion

The current study aimed to explore the relationships between dietary lipid profile, children’s eating behaviors, and screen time-related sedentary habits. The current findings demonstrate that reductions in screen time-related sedentary habits are associated with a lower intake of total fat and saturated fat. Conversely, increases in screen time-related sedentary habits correlate with the higher consumption of these dietary fats. This increased fat intake has been linked to a specific eating behavior in children, namely the “Desire to Drink”, an understanding of which can help to manage children’s eating and sedentary behaviors to improve their diets and, consequently, their overall health.

Dietary fats, including total fat and specific types like saturated and unsaturated fats, are essential components of a balanced diet and play a crucial role in children’s growth and development [38,39]. Total fat contributes to energy balance, supports cellular function, and aids in the absorption of fat-soluble vitamins [40]. A reduction in fat intake is necessary when this fat is derived from energy-dense and nutrient-poor sources, such as ultra-processed foods, which are often high in saturated fats and linked to a poor dietary lipid profile. Encouraging the consumption of nutrient-dense, whole foods, such as avocados, nuts, seeds, olive oil, and fatty fish can provide high-quality fats while supporting overall nutritional adequacy [38,40]. By promoting a diet that includes a wide variety of foods and nutrients, children can meet their energy and developmental needs while minimizing the risk associated with the excessive consumption of unhealthy fats and sugars [41].

No significant associations were found between screen time and the relevant group of omega-3 fatty acids, specifically EPA and DHA. It is possible that omega-3 intake in the studied sample was insufficient to observe relevant effects. This suggests the need for further investigation into the relationship between essential fatty acids and children’s diets in populations with higher omega-3 consumption, as well as promoting their intake as part of a healthy dietary pattern. The current results do not undermine the importance of omega-3 fatty acids, which have been shown in other research to support healthy lipid metabolism and reduce the risk of cardiovascular diseases, among other health benefits [42,43,44]. While the current study did not demonstrate this effect, it remains crucial to emphasize the value of incorporating omega-3-rich foods into children’s diets, ensuring their intake is not compromised by unhealthy lifestyle habits.

Television viewing has been associated with adverse dietary outcomes in children aged 2–6, specifically with a higher intake of energy from total fat and trans fats [45]. Additionally, sedentary behavior, in general, has been linked to a higher percentage of energy from dietary fat [46], further contributing to an increased obesity risk in children and adolescents when exposed to screen time and sedentary lifestyles [47]. A higher intake of low-quality fats and empty calories through ultra-processed food consumption, such as chips, candies, and sugar-sweetened beverages, has been associated with increased screen time in other studies [48,49,50,51]. This behavior may be driven by factors such as reduced awareness of food consumption during television viewing and the influence of advertisements promoting energy-dense, nutrient-poor products [49,51]. These foods are often high in saturated fats, particularly saturated and trans fats, contributing to unfavorable dietary lipid profiles [52]. Furthermore, prolonged sedentary behavior during screen time limits opportunities for physical activity, a key factor in maintaining energy balance [53,54].

The reduction in energy expenditure caused by decreased physical activity can lead to a positive energy balance, resulting in excessive weight gain and an increased risk of childhood obesity [47]. This condition is strongly associated with the development of metabolic and cardiovascular diseases later in life [55,56]. These findings underline the importance of minimizing screen time and encouraging healthier dietary behaviors to improve dietary lipid profiles, mitigate these risks, and promote long-term health. While our analysis highlights the associations between screen time, dietary lipid profile, and eating behavior, it is important to acknowledge that other lifestyle factors, such as physical activity and sleep, could also play a role in shaping dietary choices. Furthermore, in this study, the children’s diets were similar due to their shared cultural context and uniform healthy lifestyle recommendations. However, the impact of sedentary behavior on dietary lipid profiles may differ with broader dietary patterns. For instance, a Mediterranean diet, rich in healthy fats like olive oil and nuts, might counteract some negative effects of sedentary habits, whereas a Western diet, higher in saturated and trans fats, could worsen them [57]. These factors warrant further exploration in future studies to complement the findings presented here.

CEBQ has been widely used to assess eating behaviors in children, with research linking specific behaviors to dietary intake [58]. For example, one study found that certain eating behaviors were associated with dietary patterns and adiposity in young children [59], and another study demonstrated that appetite-related behaviors influenced children’s food intake at later ages [60]. In our study, we observed that the “Desire to Drink” domain, which specifically reflected the tendency to consume beverages other than water, such as fruit juices, sodas, and sugary drinks, was associated with higher total fat consumption, further emphasizing the role of specific eating behaviors in shaping children’s lipid profiles. Excessive consumption of sugary beverages contributes to empty calories which often goes along with the intake of ultra-processed foods and a sedentary lifestyle, and significantly increases the risk of obesity and related health conditions [48,61]. It is important to educate children appropriately to promote water as the primary beverage choice, while encouraging the very occasional consumption of other drinks [62].

This educational task is closely tied to how parents choose to feed their children. Children’s eating behaviors are significantly influenced by parental feeding practices, which include strategies such as controlling food portions, providing healthy food options, and modeling healthy eating habits [63,64,65]. Evidence suggests that positive parenting practices can promote healthier dietary choices in children, while unbalanced practices, whether due to excess, deficiency, or restriction, can lead to unhealthy eating behaviors [66,67]. The influence of parental behavior on children’s eating habits and diet has been well-documented, with most studies focusing on its impact on caloric intake, sugar, sweets, and sugary drink consumption, as well as the critical role of fruit and vegetable intake, particularly when it is low [9,44,45,46,47,48]. Parental practices were not evaluated in this study because they are already well-established factors affecting children’s eating behavior [68]. Instead, the focus was on the children’s own behaviors, such as eating habits and screen time, to assess their independent impact on lipid profiles. However, nutritional education for parents, which is then passed down to their children, could be a valuable strategy for improving lipid profile and promoting healthy habits overall.

Educational campaigns at the community and school levels play a crucial role in addressing unhealthy dietary and sedentary habits in children. These initiatives can effectively promote awareness about the benefits of reducing screen time, increasing physical activity, consuming water as the primary beverage, and incorporating nutrient-dense foods, including omega-3-rich options, into daily diets [69,70,71,72,73]. By targeting both children and their immediate environments, such campaigns have the potential to create a supportive ecosystem that sustains healthy habits and mitigates the risk of obesity and related health conditions. Our findings emphasize the importance of addressing sedentary behavior in public health policies aimed at improving dietary quality in children. Interventions targeting both reduced screen time and the promotion of balanced dietary lipid intake could have synergistic effects on reducing the risk of diet-related chronic conditions. Policies should consider culturally adapted strategies to promote healthy lifestyle behaviors, ensuring that recommendations align with the dietary patterns prevalent in the target population.

### Strengths and Limitations of the Study

The current study shows strengths, notably its longitudinal design, which allows for the examination of the temporal relationship between children’s eating behaviors, screen time, and dietary lipid profiles. The focus on independent variables, such as children’s eating habits and screen time, ensures that the findings reflect the direct effects of these factors on lipid profile. Another strength is the comprehensive assessment of dietary intake, including the measurement of essential fatty acids, which contributes to a more detailed understanding of children’s lipid profile and overall nutritional status. The use of a well-established tool, such as the CEBQ, allowed for a comprehensive assessment of children’s eating behaviors. Additionally, the study’s sample size and the inclusion of children from diverse backgrounds strengthen its generalizability and relevance to different populations. The study’s design also benefits from its focus on real-world behaviors, such as screen time and dietary habits, which are often influenced by family dynamics and environmental factors. This makes the findings particularly relevant for informing public health interventions aimed at improving children’s dietary habits and reducing sedentary behaviors.

Despite these strengths, there are some limitations. One potential limitation is the reliance on self-reported data for certain behaviors, which may introduce biases related to recall accuracy or social desirability. The study also focused on a specific set of behaviors and dietary factors, which may not capture the full range of influences on children’s lipid profile. Factors such as physical activity levels, sleep patterns, genetic background or family environment could be determinants of dietary habits in young children. Future research should consider these variables to provide a more comprehensive understanding of the topic. Moreover, future studies could benefit from exploring additional factors, such as dietary variety or other environmental influences on children’s eating habits. Although the study included a relatively large sample, the ability to generalize the screen time findings to other age groups would be the final limitation.

## 5. Conclusions

This study emphasizes the importance of managing children’s screen time and eating behaviors to improve dietary lipid profile. Current findings show that reducing screen time is linked to a lower intake of total and saturated fats, which can have positive effects on nutritional intake, and overall health. Limiting the consumption of sugary drinks and encouraging water as the primary beverage, along with reducing sedentary behaviors, are key strategies for the prevention of childhood obesity and related health conditions. Effective nutrition education for both children and parents is essential in terms of promoting healthier habits and ensuring long-term health improvements.

## Figures and Tables

**Table 1:** The sociodemographic characteristics of the sample according to changes in screen/sedentary time in 9 months.

	T1: Reduction in Screen/Sedentary Time § n = 30	T2: Without Changes in Screen/Sedentary Time § n = 30	T3: Increased Screen/Sedentary Time § n = 30	*p-*Value
n (%)				
Intervention group				
Control	16 (53.3)	15 (50.0)	14 (46.6)	0.640
Intervention	14 (46.6)	15 (50.0)	16 (53.3)	
Sex of the child				
Male	9 (30.0)	8 (26.6)	13 (46.5)	0.201
Female	21 (70.0)	22 (73.3)	17 (53.5)	
Child living with				
Mother and father	25 (83.3)	20 (66.6)	22 (73.3)	0.257
Time split between mother and father	2 (6.6)	1(3.3)	3 (10.0)	
Only mother	3 (10.0)	0 (0.0)	1 (3.3)	
Only father	0 (0.0)	0 (0.0)	2 (6.6)	
Others	0 (0.0)	9 (30.0)	2 (6.6)	
Parents’ educational level				0.308
Elementary	10 (33.3)	11 (36.6)	11 (36.6)	
2 years high school	8 (26.6)	6 (20.0)	2 (6.6)	
At least 3 years high school	6 (20.0)	6 (20.0)	7 (23.3)	
University	5 (16.6)	5 (16.6)	7 (23.3)	
Without studies	1 (3.3)	2 (6.6)	3 (10.0)	
Parents’ job situation				0.201
Working	15 (50.0)	14 (46.6)	20 (66.6)	
Unemployed	9 (30.0)	5 (16.6)	4 (13.3)	
Other	6 (20.0)	11 (36.6)	6 (20.0)	
Monthly salary				0.750
None	8 (26.6)	5 (16.6)	2 (6.6)	
Low income	4 (13.3)	3 (10.0)	5 (16.6)	
Lower-middle income	12 (40.0)	18 (60.0)	15 (50.0)	
Upper-middle income	4 (13.3)	2 (6.6)	3 (10.0)	
High income	2 (6.6)	2 (6.6)	5 (16.6)	
Mean (SD)				
Age of the child (years)	5.1 (1.3)	5.3 (1.4)	5.5 (1.3)	0.584
BMI of the child (kg(m^2^)	23.3 (3.9)	24.2 (3.6)	23.1 (3.2)	0.469

Abbreviations: SD: standard deviation. BMI: body mass index. Differences in means between groups were tested by one-way ANOVA. Differences in prevalences across groups were examined using χ^2^. §: differences in screen/sedentary time (measured by weekly screen hours) between baseline and at 9 months follow-up distributed in tertiles.

**Table 2:** Changes in screen/sedentary time separated into tertiles and related to changes dietary fat profile.

	T1: Reduction in Screen/Sedentary Time § n = 30	T2: Without Changes in Screen/Sedentary Time § n = 30	T3: Increased Screen/Sedentary Time § n = 30	*p-*Value
Mean (SD)				
Dietary total fat (g)				0.046
Baseline	59.4 (15.1)	54.3 (14.5)	70.4 (25.1)	
9 months	48.2 (13.6) ^b^	47.4 (19.5) ^c^	78.6 (41.6) ^b c^	
▲	−11.1 (10.6) * ^e^	−6.9 (21.3)	8.1 (16.5) ^e^	
Dietary SFA (g)				0.030
Baseline	25.4 (6.6)	22.3 (4.8)	26.1 (11.4)	
9 months	21.1 (5.8)	19.9 (7.6)	28.1 (15.4)	
▲	−4.3 (6.1) *	−2.4 (7.9)	2.1 (3.9) *	
Dietary MUFA (g)				0.099
Baseline	18.4 (4.7)	19.1 (5.6)	26.2 (7.2)	
9 months	16.4 (4.5) ^b^	16.2 (7.1) ^c^	28.8 (13.5) ^b c^	
▲	−2.1 (3.7)	−2.9 (7.7)	2.6 (6.3)	
Dietary PUFA (g)				0.135
Baseline	8.7 (3.9)	8.1 (4.2)	10.9 (2.5)	
9 months	6.9 (2.9) ^b^	7.1 (2.8) ^c^	12.7 (6.8) ^b c^	
▲	−1.8 (2.5)	−1.1 (3.5)	1.7 (4.3)	
Dietary cholesterol (mg)				0.258
Baseline	280.6 (91.3)	234.2 (105.3)	437.2 (100.2)	
9 months	233.4 (72.5)	218.2 (104.3)	281.4 (11.5)	
▲	−47.1 (104.5) *	−16.1 (87.8)	−155.7 (88.6)	
Dietary EPA (g)				0.831
Baseline	0.01 0.01 (0.01)	0.01 (0.02)	0.1 (0.2)	
9 months	0.04 (0.1)	0.02 (0.05)	0.1 (0.1)	
▲	0.03 (0.1)	0.01 (0.06)	−0.06 (0.3)	
Dietary DHA (g)				0.880
Baseline	0.01 0.02 (0.02)	0.04 (0.05)	0.3 (0.4)	
9 months	0.09 (0.2)	0.04 (0.1)	0.2 (0.1)	
▲	0.06 (0.2)	−0.01 (0.1)	−0.1 (0.5)	
Dietary total energy (kcal)				0.177
Baseline	1632.1 (339.9)	1574.9 (361.1)	1726.5 (513.6)	
9 months	1353.9 (321.3) ^b^	1336.7 (281.1) ^c^	1997.4 (190.9) ^b c^	
▲	−278.1 (334.5)	−238.2 (349.2)	270.8 (322.7)	
Dietary fiber (g)				0.968
Baseline	14.7 (5.9)	14.1 (3.3)	17.1 (0.1)	
9 months	14.5 (3.3)	13.4 (6.1)	16.4 (3.1)	
▲	−0.2 (5.8)	−0.6 (5.7)	−0.7 (3.1)	

Abbreviations: SD: standard deviation. SFA: saturated fatty acids. MUFA: monounsaturated fatty acids. PUFA: polyunsaturated fatty acids. EPA: eicosapentanoic acid. DHA: docosahexaenoic acid. §: differences in screen/sedentary time (measured by weekly screen hours) between baseline and at 9 months follow-up distributed in tertiles. ▲: change between baseline and 9 months. * time-related changes within the group. (b,c): letters denote group differences at each time point (ANOVA). (e): letters denote intergroup differences in time-related changes (post hoc analysis). GLM was adjusted by socioeconomic level, body mass index (kg/m^2^), weight (kg), and height (m).

**Table 3:** Pearson correlations between changes in dietary fat and changes in CEBQ domains within a 9-month period.

	*r*	*p*-Value
Food responsiveness	−0.031	0.859
Enjoyment of food	−0.193	0.265
Emotional overeating	−0.049	0.781
Desire to drink	0.528	<0.001
Satiety responsiveness	0.179	0.304
Slowness in eating	0.129	0.467
Emotional undereating	0.193	0.267
Fussiness	0.294	0.087

Abbreviations: r: correlation coefficient.

## Data Availability

There are restrictions on the availability of the data used for this trial due to the signed consent agreements around data sharing, which only allow access to external researchers for studies adhering to the project’s purposes. Requestors wishing to access the trial data used in this study can make a request to pep.tur@uib.es.
